# Cancer in children residing near nuclear power plants: an open question

**DOI:** 10.1186/1824-7288-36-60

**Published:** 2010-09-10

**Authors:** Giovanni Ghirga

**Affiliations:** 1 International Society of Doctors for Environment (ISDE) Alto Lazio, Italy

## Abstract

**Background:**

Global warming and the established responsibility of the anthropogenic emissions of greenhouse gases represent a strong push towards the construction of new nuclear power plants (NPPs) to cope with the growing energy needs. The toxicity of nuclear waste associated with the extreme difficulty of their disposal and increase in cancer mortality and incidence following occupational radiation exposure are considered the only health problems.

**Methods:**

A search of scientific articles and government documents published since January 1, 1980 to July 1, 2010 was performed to evaluate cancer rate and mortality in residents, particularly children, in the vicinity of NPPs.

**Results:**

A recent well conducted state-of-the-art case-control study of childhood cancers in the areas around German NPPs (KiKK study) showed a statistically significant cancers (2.2-fold increase in leukemia and a 1.6-fold increase in solid tumor) among children under five years of age living in the inner 5 km circle around NPPs when compared to residence outside this area. These findings have been confirmed by two meta-analyses. Nevertheless, other UK, France, Spain and Finland studies did not find cancer incidence and/or death increase near NPPs.

**Conclusions:**

Increased cancer risk near NPPs remains in fact an open question. The stronger evidence from the KiKK study suggests there may well be such increases at least in children regardless of the country in which nuclear reactors are located. In fact, few months ago the U.S. Nuclear Regulatory Commission has asked the National Academy of Sciences (NAS) to perform a state-of-the-art study on cancer risk for populations surrounding NPPs.

## Background

Global warming and the established responsibility of the anthropogenic emissions of greenhouse gases represent a strong push towards the construction of new nuclear power plants (NPPs) to cope with the growing energy needs. Such a view however is based on the premise that nuclear power represents a safe, clean, sustainable and economic alternative and only nuclear waste could be a "solvable" problem.

Nevertheless, scientific data show a different view. Along with an increase in cancer mortality and incidence following occupational radiation exposure, health consequences from populations residing near NPPs are a controversial issue.

## Methods

A search of scientific articles (PubMed/EMBASE) and government documents published since January 1, 1980 to July 1, 2010 was performed to evaluate cancer rate and mortality in residents, particularly children, in the vicinity of nuclear power plants (NPPs).

## Results

In the late 1980 s and early 1990 s, increased incidence of childhood leukemia were reported near United Kingdom nuclear facilities but the cause or causes remained unknown because it was estimated that the radiation doses from these facilities were too low to explain the increased leukemia [1-4].

In Germany, since a childhood leukemia cluster was first reported in 1980 in the vicinity of the nuclear plant Krümmel near Hamburg public anxieties have remained high [5,6].

In 2002 the German government contracted with the Childhood Cancer Registry at the University of Mainz (GCCR) to conduct a state-of-the-art case-control study of childhood cancers, particularly leukemia, in the areas around the country's 16 commercial nuclear power plants (NPPs). This Epidemiological Study on Childhood Cancer in the Vicinity of Nuclear Power Plants (Epidemiologische Studie zu Kinderkrebs in der Umgebung von Kernkraftwerken) is known by the acronym KiKK [7,8]. In contrast to ecological studies that compare geographic averages of disease rates at area mid-point distances from the suspected source, a case-control study compares characteristics of individual children who suffer from disease (cases) with those of the same age and sex who live in the same area and do not suffer from this disease (controls). In the KiKK study, researchers determined the distances of the places of residence of cases (at the time of diagnosis) and of controls with an accuracy of within 25 m. Thus a possible distance dependency of cancer risk could be determined with much greater reliability than in ecological studies.

In order to lend maximum credibility to this investigation, the German Federal Office for Radiation Protection (Bundesamt für Strahlenschutz, BfS) appointed an independent external review committee of 12 scientists (5 epidemiologists, 2 pediatricians, 2 statisticians, and 3 physicists) to assist in the study's design and evaluation.

The KiKK study examined all cancers at all 16 nuclear reactor locations in Germany between 1980 and 2003, including 1,592 patients under five years of age with cancer (excluding leukemia) and 4,735 controls, with 593 under five years of age with leukemia and 1,766 controls. This means that the study is statistically strong and its findings statistically significant. Small numbers and weak statistical significance often limit the usefulness of many smaller epidemiological researches.

This study showed a statistically significant cancers (2.2-fold increase in leukemia and a 1.6-fold increase in solid tumor, mainly embryonal), among children under five years of age living in the inner 5 km circle around nuclear power plants when compared to residence outside this area. Risks for leukemia were also elevated in the 10 km zone. Regression models investigating the distance of residence at time of diagnosis to the nearest nuclear power plant showed a significant distance trend.

Subsequent to the publication of the KIKK study, the Federal Minister for the Environment, Nature Conservation and Nuclear Safety charged the Commission on Radiological Protection with the Evaluation of the Study. In September 2008, the Commission on Radiological Protection submitted its Statement: "This study confirms a government-sponsored study of childhood cancer in the proximity of German nuclear power plants (KiKK) found that children < 5 years living < 5 km from plant exhaust stacks had twice the risk for contracting leukemia as those residing > 5 km" [9].

In 2009, the Commission on Radiological Protection Presented a Substantiation of Its Statement to the Scientific Community [10].

The KiKK's findings have been confirmed by two meta-analyses. Baker and Hoel analyzed data from 17 research papers world-wide covering 136 nuclear sites in the UK, Canada, France, the United States, Germany, Japan and Spain [11]. In children up to nine years old, leukemia death rates were from 5 to 24 per cent higher, and leukemia incidence rates were 14 to 21 per cent higher. These findings were statistically significant. Körblein carried out a meta-analysis of leukemia near most NPPs in Germany, France and the UK [12]. He also found a statistically significant increased risk of child relative risk of leukemia deaths in residents in the surrounding areas of nuclear reactors.

Previous UK [13,14] and French [15,16] studies have not found evidence of leukemia increases, apart from one UK study [17]. However, since the KiKK study was published, more recent studies have suggested small increases may exist, albeit without statistical significance [18,19].

The words "no evidence of an excess risk of leukemia" in the conclusions of the papers by Evrard et al. [20] and Bithell et al. [21] as well as Laurier et al.'s statement [19] are often misleadingly interpreted as negative rather than as inconclusive findings. These interpretations ignore a fundamental rule in epidemiology: "absence of evidence of an effect does not constitute evidence of absence of that effect" [22]. Clearly, studies that are inconclusive due to low statistical power or flawed design cannot invalidate positive findings in studies with a high statistical power, such as the KiKK study.

A report carried out at the request of Spain's Congress has shown no evidence of increased risks of death for cancer due to proximity to Spanish nuclear facilities (which include both NPPs and nuclear fuel storage facilities) [23]. Nevertheless, an increase risk was observed in residents in the surrounding areas of some facilities but the authors underline that this observation did not repeat near other similar NPPs and was not statistically significant.

A study in Finland investigated leukemia incidence in children living near the two Finnish NPPs using both cohort and case-control analysis. The results were consistent and neither of them indicated an increased risk of leukemia, even though the small sample size and lack of population residing within the 5-km radius limit the strength of the conclusions [24].

## Conclusions

The French, English, Spain and Finland studies may mislead members of the public into thinking that there are no increased cancer risk near NPPs when in fact ***the question remains open***. The stronger evidence from the KiKK study suggests there may well be such increases at least in children regardless of the country in which nuclear reactors are located.

The U.S. Nuclear Regulatory Commission has asked the National Academy of Sciences (NAS) to perform a state-of-the-art study on cancer risk for populations surrounding nuclear power facilities. The study can begin this year [25].

Because it was estimated that the radiation doses from nuclear facilities were too low to explain the increased leukemia, most researcher did not investigate the possibility of radioactive contamination of the environment by the NPPs. The latter are large plants with contact to the soil via water; they have high chimneys, and many people go in and out all the time. There are multiple opportunities for radioactive contamination of the ground water, the air, and the people who work there or live in their proximity. The possible cause of the increased number of cases of leukemia may not be ionizing radiation only, but also the environment of the power station. Through water and precipitation, radioactivity reaches the food chain and even small traces could be sufficient to trigger leukemia in a toddler [26].

Among various hypotheses to explain cancer increases near nuclear installations the evidence that sensitivity to radiation is much higher during early embryonic and fetal stages [27-33] appears to be the most interesting.

Estimated radiation doses to adults near nuclear power stations are invariably very low (10^-2 ^to 10^-4 ^mSv per year). Each model derives a range of results log-normally distributed from which only the median value is normally used. This means that, although the real value could be larger or smaller than the median value, in practice some high values could result [34,35].

The cumulative uncertainty in dose estimates could be very large as recognized by the report of the UK Government's CERRIE Committee [36]. This does not mean that official dose estimates from NPPs releases are always incorrect. But it does mean they contain unquantified uncertainties which could be large and which render them unreliable where evidence exists that there may be and increate risk of cancer near NPPs.

Spikes in the emissions of radioactive carbon and hydrogen (as carbon dioxide and water vapour) occur at nuclear power reactors when their pressure vessels are opened (approximately once a year) to replace nuclear fuel [37]. These spikes in releases from nuclear power stations may result in the labelling of the embryos and fetuses of pregnant women living nearby at high concentrations. These concentrations could be long-lived and could result in high doses to radiosensitive tissues and subsequent cancers.

It has been shown that in both human and murine hematopoeitic stem cells, heritable radiation-induced changes can be seen in the first progeny. However, a significant proportion of cells that survive radiation exposure may appear normal but have mutational changes in their progeny due to genomic instability. This chromosomal instability has been causally linked to leukemia [38].

Epigenetics, a field at the epicenter of Modern Medicine [39], is modifications of the DNA or associated proteins, other than DNA sequence variation, that carry information content during cell division; nongenetic factors cause the organism's genes to express themselves differently [40]. Environmental exposures early in development play a role in gene expression or disease susceptibility later as an adult.

Epigenetics may play an important role in genomic instability caused by radiation exposure [38]. In 2008, the National Institutes of Health announced that $190 million had been earmarked for epigenetics research over the next 5 years. Government officials noted that epigenetics has the potential to explain mechanisms of aging and human development and the origins of cancer, heart disease, mental illness, as well as other conditions.

Until new data will be available on cancer risk near NPPs, the precautionary principle should be applied and radiation exposition of embryos, fetuses, infants and toddlers should be avoided even at very low doses. The precautionary principle covers cases where scientific evidence is insufficient, inconclusive or uncertain and preliminary scientific evaluation indicates that there are reasonable grounds for concern that the potentially dangerous effects on the environment, human, animal or plant health may be inconsistent with the high level of protection chosen by the E.U. [41].

In view of the Italian Government will to built new NPPs, Italian pediatricians should be informed that a possible increase of cancer risk in children residing near the future sites of NPPs cannot be excluded. They should propose to the Ministry of Health to start monitoring cancer incidence for exposed population.

For in deep commentary on NPPs and cancer in children residing nearby refer to the following articles authored by Ian Fairlie [42-44].

## Competing interests

The author declares that they have no competing interests.
